# The error‐related negativity as a neuromarker of risk or resilience in young children

**DOI:** 10.1002/brb3.2008

**Published:** 2020-12-22

**Authors:** Jamie M. Lawler, Jessica Hruschak, Kristin Aho, Yanni Liu, Ka I. Ip, Renee Lajiness‐O’Neill, Katherine L. Rosenblum, Maria Muzik, Kate D. Fitzgerald

**Affiliations:** ^1^ Eastern Michigan University Ypsilanti MI USA; ^2^ Wayne State University Detroit MI USA; ^3^ University of Michigan Ann Arbor MI USA; ^4^ Yale University New Haven CT USA

**Keywords:** anxiety, cognitive control, emotion regulation, error‐related negativity, executive functioning

## Abstract

**Introduction:**

The error‐related negativity (ERN) is a neural response that reflects error monitoring. Contradictorily, an enlarged (more negative) ERN has been cited as both a risk factor and a protective factor, which hinders its utility as a predictive indicator. The aim of the current study was to examine the associations between ERN measured in early childhood with the development of cognitive control (CC), emotion regulation, and internalizing/externalizing symptoms over 1–2 years.

**Methods:**

When children were ages 5–7, EEG was collected during a Go/No‐Go task. A subset of the original participants (*n* = 30) were selected based on their baseline ERN in an extreme‐case design: half with high‐amplitude ERN, matched by age and sex with another group with low‐amplitude ERN.

**Results:**

At follow‐up, children in the High‐Amplitude group showed better executive function, less self‐reported anxiety and depression, less affect dysregulation, more parent‐rated CC, less lability/negativity, and fewer parent‐reported externalizing problems. Many results held even when accounting for baseline levels. Further, emotion dysregulation mediated the relationship between the ERN and both anxiety and externalizing problems, while CC mediated the ERN's relationship with externalizing problems only.

**Conclusions:**

These results can inform identification and intervention efforts for children at risk for psychopathology.

## Graphical abstract

High‐amplitude error‐related negativity (ERN), measured in early childhood, predicts better self‐control, including executive function and cognitive control and less affect dysregulation in middle childhood, and predicts fewer anxiety, depression, and externalizing symptoms over time. Emotion dysregulation mediated the relationship between the ERN and both anxiety and externalizing problems, while cognitive control mediated the ERN's relationship with externalizing problems only.

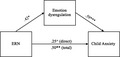

## 1 INTRODUCTION

Mental health disorders occur at alarmingly high rates in children (~20%; Kashani & Orvaschel, 1990) and are associated with chronic trajectories of illness (Jaffee et al., 2002). Strategies to prevent worsening trajectories of early mental health problems are urgently needed, but will depend on the identification of factors that predict divergent pathways from early life risk to either persistent psychopathology or mental wellbeing. One potential neuromarker of risk for psychopathology is the error‐related negativity (ERN), a neural response that reflects error monitoring processes (Gehring et al., 2012). Contradictorily, an enlarged (more negative) ERN has been cited as both a risk factor and protective factor (Weinberg et al., 2015). A larger ERN predicts higher risk for anxiety disorders (e.g., Meyer et al., 2013), but also has been associated with greater cognitive control abilities (Larson & Clayson, 2011). In turn, cognitive control abilities predict fewer externalizing and internalizing symptoms in children over time (Ip et al., 2019; Lengua, 2006). Emerging evidence discussed below suggests that there may be a developmental shift in the relationship between ERN and anxiety, perhaps related to cognitive control, with early childhood being a particularly salient point in time (Ip, Liu, et al., 2019; Moser, 2017; Moser et al., 2015). The aim of the current study is to examine the associations between brain activity (ERN) measured in early childhood with the development of cognitive control and internalizing and externalizing symptoms over 1–2 years.

### 1.1 Error‐related negativity and anxiety

The ERN is a negative deflection that occurs approximately 50 ms after an individual makes a mistake (Falkenstein et al., 1991; Gehring et al., 1993) and reflects neural activity in the anterior cingulate (ACC) region (Mathalon et al., 2003). Numerous studies have shown that a higher amplitude ERN is associated with anxiety in older children and adults, both continuously (Hajcak et al., 2003) and when predicting clinical disorder (Ladouceur et al., 2006; Meyer et al., 2017). Even in young children (age 6), a heightened ERN predicted anxiety disorders concurrently (Meyer et al., 2013) and over time (Meyer et al., 2015). However, mounting evidence suggests that anxiety is *inversely* related to ERN amplitude when examined continuously in children ages ~10 and younger (Ip, Liu, et al., 2019; Lo et al., 2017; Meyer et al., 2012; Moser et al., 2015; Torpey et al., 2013). Meyers (2017) suggests these seemingly conflicting findings may indicate a developmental shift in the *content* of children's anxiety, leading to the inverse brain activity patterns. Young children, she argues, tend to be fearful of external stimuli (e.g., the dark), while older children tend to be more fearful of internally generated stimuli (e.g., their performance on a task). Highly anxious young children may then be preoccupied with the environmental conditions of the task (typically in a relatively dark room, with a stranger, wearing an unfamiliar cap), while anxious older children react to the mistakes made on the task. In Meyers' framework, she suggests that young children with *clinically significant anxiety* are operating at a more “mature” level in terms of the content of their anxiety (responding to internally generated stimuli, i.e., their performance on a task) and thus show the increased ERN pattern. A recent study by Meyer et al. (2018) tested this hypothesis and found that children who were fearful in toddlerhood showed a smaller ERN at age 6, but that fearfulness in toddlerhood and blunted ERN at age 6 predicted a larger ERN at age 9. Despite this valuable finding, Meyers and colleagues did not examine the behavioral or clinical sequelae of a larger or smaller early ERN to determine whether the ERN is useful in predicting risk for later anxiety symptoms or other psychopathology. Further, they did not investigate the relationship with objective measures of cognitive control, a critical construct for the expression of emotional and behavioral problems, including anxiety. The current study will address this gap in the literature by examining the predictive value of ERN measured in early childhood for parent‐ and self‐reported psychopathology as well as performance on objective measures of cognitive control capacity.

### 1.2 Error‐related negativity and cognitive control

A higher amplitude ERN has also been associated with better cognitive control (Larson & Clayson, 2011), which is defined as capacity to resolve conflict between competing response options and to inhibit pre‐potent, but inappropriate, responses to achieve task goals. Errors during task performance elicit the ERN, which has been understood to index signaling for increased cognitive control (Falkenstein et al., 2000). Moreover, individuals with greater working memory capacity tend to show a larger ERN than individuals with a lower working memory capacity (Miller et al., 2012), suggesting that a larger ERN may related to better executive function generally. Similar results have been found in children, where higher levels of attentional control were associated with a larger ERN/Correct‐related negativity (CRN) difference score (∆ERN) at 10–15 years old (Samyn et al., 2014). Torpey et al. (2012) similarly found that a larger ∆ERN was associated with better behavioral inhibitory control performance on a go/no‐go task in 5‐ to 7‐year‐olds. Additionally, Meyer and Klein (2018) found that greater parent‐rated cognitive control was related to a larger ERN in 6‐year‐old children. In one short‐term longitudinal study of 4‐ to 6‐year‐olds, Grammer et al. (2018) showed that larger ERN predicted better attention control 6 months later. The current study will extend these findings to examine predictive associations over at 1‐ to 2‐year period with several measures of cognitive control.

### 1.3 Cognitive control, anxiety, and the ERN

In both cross‐sectional and longitudinal studies, greater capacity for cognitive control is associated with fewer internalizing symptoms, including anxiety, in children (Eisenberg et al., 2009; Lengua, 2003, 2006; Nelson et al., 2018). Thus, cognitive control should be protective against anxiety; however, the relation of each to the ERN appears to create a paradox. Relatively few studies have examined anxiety, cognitive control and the ERN in the same subjects, particularly children. One such study of 6‐year‐old children by Meyer and Klein (2018) utilized parent report of cognitive control and shyness and found that both were related to an increased ERN, while fearfulness was associated with a decreased ERN. They found that the ERN was increased in children with anxiety disorders, and that this association was explained by shyness, but not fear or cognitive control. In contrast, the ERN was blunted in children with externalizing disorders (ADHD or ODD), and this association was accounted for by lower levels of both shyness and cognitive control. While this study provided some insight regarding the relationships between the ERN, cognitive control, and anxiety, it is limited by using only parent report of the two latter constructs, as well as a cross‐sectional design. The current study will address these limitations by utilizing objective, behavioral measures of cognitive control, child‐report on anxiety and by examining relationships with the ERN across time.

Further, past research relates cognitive control to the overlapping, but distinct construct of emotion regulation (Zelazo & Cunningham, 2007). Emotion regulation is the processes by which emotional reactions are modulated to achieve individual goals (Thompson et al., 2008). Cognitive control allows an individual to direct attention away from emotionally salient stimuli, to think flexibly to consider alternate interpretations, and to inhibit undesirable emotional displays. Some work suggests that reduced capacity for emotion regulation may be important to the expression of childhood anxiety and may account for cognitive control‐anxiety associations (Ip, Jester, et al., 2019). Yet, no prior studies have examined the relationship between the ERN and emotion regulation *per se* in children. The current study will extend prior work by examining measures of emotion regulation, in addition to cognitive control, to better understand pathways linking the ERN to the course of anxiety from early to middle childhood.

### 1.4 Current study

The purpose of this study is to elucidate the predictive value of the ERN in early childhood. Using an extreme group design (Preacher et al., 2005) and a longitudinal follow‐up, we examine differences in cognitive control, emotion regulation and anxiety in middle childhood between children with large and small ERN amplitudes measured in early childhood. Given past research, we expect that children characterized by a large amplitude ERN in early childhood, compared with the low‐amplitude ERN group, will show better cognitive control and emotion regulation and fewer anxiety symptoms at follow‐up in middle childhood. We will also examine the predictive utility of the ERN measured in early childhood for forecasting other internalizing (i.e., depression) and externalizing symptoms. Finally, we will explore whether cognitive control and emotion regulation mediate the relationship between the early childhood ERN and later symptoms.

## 2 MATERIALS AND METHODS

The children in this study were part of a broader project examining behavioral and neurobiological markers of childhood depression and anxiety and were sampled to represent the continuum for risk for psychopathology. Approximately one to 2 years after their initial laboratory visit, selected participants were invited to take part in a follow‐up study of cognitive control and psychopathology. This study was approved by the University Institutional Review Board.

### 2.1 Participants

The original study recruited 80 children (45 girls) from the community and from a University Psychiatry Clinic to capture the full spectrum of symptom severity. Participants were 4–7 years old at the time of the baseline assessment (*M* = 5.8, *SD* = 1.11). Parents reported child race/ethnicity as 65% Caucasian, 11.3% African American, 1.3% Latino, 2.5% Asian, and 20% biracial. To be eligible for participation, children had to be between 4.00 and 7.99 years old and have no history (by parent report) of significant neurodevelopmental delay (autism spectrum disorder or cognitive impairment), serious medical illness, or head injury, and no exposure to medications that affect central nervous system.

#### 2.1.1 Subsample selection

From this initial group, a subset of study participants (*n* = 30, 60% male) were selected based on their baseline ERN in an extreme‐case design: one group of children with large amplitude (more negative) ERN (*n* = 15), matched by age and sex (when possible) with a second group with small amplitude (less negative) ERN (*n* = 15). This matching was to ensure equal distributions of ages in each group given past research demonstrating the relation of ERN to age (Meyer, 2017) and gender (Ip, Liu, et al., 2019). Two of the 15 dyads were unable to be matched by sex. In one case, a data entry error resulted in a child's sex being misclassified at baseline. In the other case, no sex‐matched participant was available in the appropriate age range. To be eligible for selection, children needed valid ERN data at baseline and had to be at least 7 years old at follow‐up (for age‐appropriateness of self‐report measures). ERN measured at FCz (mid‐frontal EEG electrode) was used to select groups (see details below on ERN collection and processing). To select and match participants, children were split into quintiles based on ERN amplitude. Then, children in the two high‐amplitude quintiles were matched to children from the two low‐amplitude quintiles. Each matched pair was selected so that the two children were as close in age at baseline as possible, with a difference no larger than 6 months. For the subset of 30 children, mean age at baseline was 6.7 years (*SD *= 0.70) and mean age at the follow‐up was 8.3 years (*SD *= 0.71). Additional participant characteristics for the 30 follow‐up participants are found in Table 1.

**Table 1:** Participant characteristics

	High‐amplitude ERN *n* = 15	Low‐amplitude ERN *n* = 15	Overall *n* = 30
	*M* (*SD*)	*M* (*SD*)	*M* (*SD*)
	range	range	range
*Child age (months)*			
Baseline	80.1 (7.6)	80.1 (9.3)	80.1 (8.4)
	67–92	68–95	67–95
Follow‐up	99.1 (8.5)	100.1 (8.8)	99.6 (8.5)
	88–117	86–114	86–117
*Length of Follow‐up (months)*	19.1 (6.1)	19.9 (4.6)	19.4 (5.4)
	11–32	14–27	11–32
	Percent	Percent	Percent
*Sex* ^a^			
Male	67	53	59
Female	33	47	41
*Child race*			
White/Caucasian	60	47	53
Black/African American	20	13	17
Asian or Pacific Islander	7	0	3
Biracial	13	40	27

^a^ There were no significant differences between males and females on any of the study outcomes (*p's* > 0.1)

### 2.2 Procedure overview

#### 2.2.1 Baseline session

A phone screen was completed to determine initial eligibility. Participants and their parents then came to the laboratory where parents completed written consent and children provided oral assent. Following consent/assent, research assistants brought children to a child‐friendly EEG suite while parents filled out questionnaires. Following EEG cap application, ERP data were collected during a child‐friendly Go/No‐Go task (see measures below). Parents also completed questionnaire measures of cognitive control and internalizing/externalizing symptomatology. Additional non‐computerized measures were completed with the child (e.g., tasks from the Laboratory Temperament Assessment Battery [Goldsmith et al., 1999] to measure positive and negative valence) and a diagnostic interview, yielding dichotomous diagnoses, was completed with the parent; however, these measures are not reported here.

#### 2.2.2 Follow‐up session

The selected subset of participants completed follow‐up data collection at a home‐visit approximately one to 2 years (*M* = 19.5 months, *SD* = 5.4 months, range = 11–32 months) following their baseline session. A trained research assistant arrived at the participant's home and completed parental consent and child assent procedures. Research assistants led participants through a number of tablet‐based tasks to assess their cognitive control abilities. Research assistants read child self‐report items out loud to participants to complete measures of mental health symptoms. Children received small toy prizes for participation. Parents also reported on their child's executive functioning, emotion regulation, and emotional and behavioral problems. See measures below. Families were compensated for their time.

### 2.3 Measures at baseline

#### 2.3.1 ERN task

The child‐friendly Go/No‐Go “Zoo” task (Grammer et al., 2014; McDermott, 2005) was used to elicit the ERN. In the Zoo task, children were asked to help a zookeeper return loose animals to their cages, except three friendly orangutans who are the zookeeper's “helpers” and should remain free. Children were asked to put the loose animals back in their cages by pressing a button as quickly as they could every time an animal picture was presented (Go Trials), but to withhold their response each time they saw an orangutan (No‐Go trials).

Children completed 8 blocks of the task, each including 30 Go trials and 10 No‐Go trials for a total of 320 trials. For each trial, a fixation cross was presented for 200–300 milliseconds (ms), followed by an animal image presented for 750 ms, and a blank screen for 500 ms. Responses could be made during the animal image and blank screen presentation. Each block consisted of novel sets of animal images, balanced on color, animal type, and size. The task was presented using Eprime software (Psychology Software Tools, Inc.). Before the experimental trials of the Zoo task, children practiced on a set of 12 trials, 3 with orangutans, and 9 with other animals and could practice multiple times until they understood the task.

#### 2.3.2 EEG set‐up and processing

The EEG was recorded using EEGLAB from 18 Ag/AgCI scalp electrodes (10/20 system) and two mastoid electrodes, using BioSemi ActiveTwo recording system. Electro‐oculogram (EOG) data were recorded from electrodes placed above and below the right eye and at the outer canthi of both eyes to capture vertical EOG and horizontal EOG, respectively. Data were referenced to a ground formed from a common mode sense active electrode and driven right leg passive electrode (see http://www.biosemi.com/faq/cms&drl.), and sampled at 1,024 Hz. Additional details can be found in Ip, Liu, et al., 2019.

For analysis, EEG data were referenced to averaged mastoid electrodes, and band‐pass filtered 0.05–30 Hz using zero‐phase shift Butterworth filters. EEG data were screened using automated algorithms that rejected epochs in which the absolute voltage range exceeded 500 μV for midline channels (Fz, FCz, Cz, and Pz), consistent with prior work (Grammer et al., 2014). Ocular movement artifacts were then corrected using a regression‐based algorithm (Gratton et al., 1983). After ocular correction, individual trials were visually inspected and rejected if any amplitudes were greater than 100 μV, differed by more than 50 μV from the previous time point, or were less than 0.5 μV in magnitude in any midline electrode, consistent with prior work (Grammer et al., 2014; Ip, Liu, et al., 2019; Perry et al., 2016).

During the Go/No‐go “Zoo” task, response‐locked ERP components were quantified using mean amplitude measurements relative to a pre‐response baseline −200 to −100 ms, consistent with prior work in young children (Grammer et al., 2014; Ip, Liu, et al., 2019). The mean amplitude of the ERN was computed for commission errors in a window 0–50 ms after the incorrect button response on No‐Go trials (Grammer et al., 2014). As in previous work (Grammer et al., 2014; Ip, Liu, et al., 2019), ERN could not be computed for omission errors (incorrect Go trials) because in these cases, there is no button response with which to link the time window. For No‐Go trials, ERN was measured at Fz (mean amplitude: −2.33 ± 5.76), FCz (mean amplitude: −3.27 ± 5.28), and Cz (mean amplitude: −1.82 ± 5.30). Overall amplitude at each of these locations was more negative on error relative to correct trials measured in the same time window (i.e., ERN effect, *p*'s < .001). As with prior work in this age group (Grammer et al., 2014; Ip, Liu, et al., 2019), ERN at FCz had the highest mean amplitude; thus, ERN measured at FCz was used to select the follow‐up groups and for mediational analyses.

#### 2.3.3 Cognitive control/Parent report

Parents completed the Child Behavior Questionnaire (CBQ; Ahadi et al., 1993; Rothbart et al., 2001). This scale consists of 195 items to assess child temperament. In order to correspond with scales completed at follow‐up, the two most theoretically and empirically salient components of effortful control: inhibitory control and attentional focusing (Rothbart et al., 2006), were combined as a baseline indicator of child cognitive control. The range of possible scores was 2–14, with higher scores indicating higher levels of cognitive control.

#### 2.3.4 Negative affectivity/Parent report

Select subscales from the CBQ were used to index emotion dysregulation and negative affectivity at baseline consistent with prior literature. Subscales included: anger/frustration, sadness, fear, discomfort, and soothability (reversed). These scales were summed into a baseline negative affectivity composite. The range of possible scores was −3 to 27, with higher scores indicating more negative affectivity.

#### 2.3.5 Internalizing and externalizing symptoms/Parent report

Given children's young age, parents reported on children's symptomatology at baseline. Parents completed the Child Behavioral Checklist (CBCL) with two possible versions based on child age at baseline: CBCL for ages 1.5–5 (Achenbach & Rescorla, 2000) or CBCL for ages 6–18 (Achenbach & Rescorla, 2001). The CBCL is comprised of 113 items that measure aspects of the child's behavior across the past 6 months. Items are rated using a three‐point rating scale (not true, somewhat or sometimes true, very often or always true). T‐scores were calculated based on published norms and then combined across measures, with higher scores indicating greater problems. The anxiety/depression subscale and the internalizing and externalizing symptom composite scores were examined.

### 2.4 Measures at follow‐up

#### 2.4.1 Cognitive control/Behavioral

Behavioral capacity for cognitive control was measured using selected subtests from the National Institutes of Health (NIH) Toolbox Cognitive Function Battery (Zelazo et al., 2013).

##### NIH Toolbox Dimensional Change Card Sort (DCCS) Test (cognitive flexibility and attention)

In this computerized DCCS task normed for ages 7–17, pictures are presented on the tablet and vary along two dimensions (shape and color). The dimension for sorting is indicated by a cue word on the screen and participants are asked to select the matching stimuli. Practice trials (four trials for each dimension) are followed by the pre‐switch block (five trials). The post‐switch block (five trials) requires sorting by the second dimension. The mixed block (40 trials) includes shifting between sorting dimensions. An age‐corrected standard score (*M* = 100, *SD* = 15) is calculated by the program based on participant accuracy and reaction time.

##### NIH Toolbox Flanker task (attention and inhibitory control)

This computerized Flanker task is presented on a tablet and asks participants to focus on a given stimulus while inhibiting attention to stimuli flanking it. In this task, participants were presented with a row of five stimuli (either fish or arrows) and pressed one of two buttons indicating the direction the middle stimulus (either a fish or arrow) is pointing. During congruent trials, all the stimuli are pointing the same direction while in the incongruent trials the flanking stimuli are pointing the opposite direction from the middle stimulus. The Flanker task included three blocks: practice (four trials), fish (20 trials), and if accuracy meets or exceeds 90%, arrows (20 trials). An age‐corrected standard score (*M* = 100, *SD* = 15) is calculated by the program based on participant accuracy and reaction time.

##### NIH toolbox list sorting test (working memory)

This List Sorting task measures the child's ability to store increasing amounts of information in working memory and accurately recall and sequence different visually and orally presented stimuli. Pictures of different foods and animals were presented along with audio recordings of the name of the object; participants were asked to say the items back in size order from smallest to largest, first within a single dimension (i.e., food or animals) and then on two dimensions (i.e., food then animals). An age‐corrected standard score (*M* = 100, *SD* = 15) is calculated by the program based on participant accuracy.

#### 2.4.2 Cognitive control/Parent report

In addition to behavioral measures of cognitive control, parents' reports on measures of cognitive control/temperament and executive function capacity were collected.

##### Child behavior questionnaire

Parents completed selected portions of the Child Behavior Questionnaire (CBQ, Ahadi et al., 1993; Rothbart et al., 2001) at follow‐up. Questions were selected to capture the two most theoretically and empirically salient components of effortful control (Rothbart et al., 2006): inhibitory control and attentional focusing. Again, the range of possible scores was 2–14, with higher scores indicating higher levels of cognitive control. This portion of the CBQ was repeated at follow‐up because past research has shown less consistency over time in this domain compared to other temperamental domains (Neppl et al., 2010).

##### Behavior Rating Inventory for Executive Function (BRIEF)

Parents reported on their child's executive function abilities on the BRIEF. This 86‐item questionnaire assesses the executive functions of youth 5–18 years of age (Gioia et al., 2000). It yields T‐scores based on published norms for: Behavioral Regulation Index (BRI), Metacognition Index (MI), and Global Executive Composite (GEC). Higher scores indicate greater problems.

#### 2.4.3 Emotion regulation/Dysregulation

Parent and self‐report was used to measure child emotion regulation/dysregulation at follow‐up. These measures were selected due to their specificity of the constructs measured. CBQ items related to negative affectivity were not repeated at follow‐up due time constraints and the stability in this domain of temperament over time (Neppl et al., 2006).

##### Emotion regulation checklist

Parents completed the Emotion Regulation Checklist (ERC, Shields & Cicchetti, 1997), a 24‐item questionnaire designed to investigate children's experience of negative or unstable mood, as well as their ability to regulate their emotions over the course of the previous week. It yields two subscales: emotional lability/negativity (higher scores indicate greater lability/negativity problems) and emotion regulation (higher scores indicate greater capacity for emotion regulation).

##### Affect dysregulation scale

Children completed the Affect Dysregulation Scale (Brown et al., 2012), a brief, six item measure which yields a total score for affect dysregulation. Example items include “In the PAST 3 MONTHS, I have felt overwhelmed by big feelings” and “In the PAST 3 MONTHS, small problems got me very upset.” Each item is rated on a Likert scale of “Not at all,” “A little,” “Sometime,” and “Often.” Total possible score range was 0–18, with higher scores indicating greater difficulties regulating affect.

#### 2.4.4 Internalizing and externalizing symptoms

Parents and children both reported on the child's symptoms. Parents completed paper questionnaires independently while children completed self‐report forms with research assistants reading questions out loud.

##### Child behavior checklist

Parents completed the CBCL (for ages 6–18; Achenbach & Rescorla, 2001) again at the follow‐up. The anxiety/depression subscale and the internalizing and externalizing symptom composite T‐scores were examined.

##### Child depression inventory

Children also self‐reported their depression symptoms on the Child Depression Inventory‐2 Self‐Report, Short (CDI‐2:SR[S]; Kovacs, 2011). The CDI‐2:SR[S] is a 12 item assessment of depression symptoms in children ages 7–17 years old. Each symptom is presented as a series of three phrases, and participants were asked to select the phrase that best represents how they feel (e.g., “I am sad once in a while”/”I am sad many times”/”I am sad all the time”). Total possible score range is 0–24 (clinical cut off score = 7–8) with higher scores indicating more depression symptoms.

##### Screen for child anxiety and related disorders

Children reported on their anxiety symptoms on the Screen for Child Anxiety‐Related Disorders (SCARED, Birmaher et al., 1999). The SCARED is a 41 item inventory rated on a 3 point Likert‐type scale that measures common symptoms of anxiety in children. Total possible score range is 0–82 (clinical cut off score = 25) with higher scores indicating more anxiety symptoms.

### 2.5 Missing data

Of the 30 participants at follow‐up, 3% were missing data on the Flanker task and the DCCS task, 10% were missing data on the List Sorting task, 3% were missing baseline CBCL data, and 3% were missing baseline CBQ data. Missing data were due to child refusal, technological failure, or experimenter error. Little's missing completely at random (MCAR) test was not significant (*χ* ^2^ = 0.344, *df* = 78, *p* = 1.000), indicating that data were missing at random. Multiple imputation was conducted in SPSS to account for missing data. Twenty imputations were conducted and analyses were completed on the pooled data (Little & Rubin, 2002).

### 2.6 Data analysis plan

Descriptive statistics, including correlations between study variables, are presented. Then, paired *t* tests (within each matched dyad) were conducted to examine differences between groups at baseline and at follow‐up. Next, to account for baseline differences between groups, in order to examine the predictive utility of the ERN over time, a two step process was used. First, a set of regression analyses were conducted predicting follow‐up outcomes from baseline proxies. For each cognitive control outcome variable (DCCS; Flanker; List Sort; parent‐reported cognitive control, executive function), parent‐reported cognitive control at baseline (CBQ attention and inhibitory control) was regressed on to the outcome variable and residuals were saved as a new variable. These residuals can be conceptualized as the construct at follow‐up controlling for baseline, or in cases where identical measures were given at both time points, they can be thought of as change in the construct over time. The same was completed for emotion regulation/dysregulation‐related variables (parent‐reported emotion regulation, lability/negativity, child‐reported affect dysregulation) with parent‐reported negative affectivity at baseline (CBQ composite), and for internalizing/externalizing variables (child‐reported depression and anxiety, and parent‐reported anxiety, internalizing, and externalizing problems), with the corresponding parent‐reported CBCL scale at baseline. Then, in order to test differences between paired groups at follow‐up, controlling for baseline, paired sample *t* tests were conducted on the residuals for each outcome variable.

Finally, mediation analyses were completed with the PROCESS macro (Hayes, 2017) for SPSS. Bootstrapped (10,000) coefficients were used to determine the significance of the indirect effect of the ERN on anxiety and externalizing symptoms through cognitive control and emotion dysregulation (separately). A cognitive control composite was used for mediation analyses to limit the number of statistical tests and thus Type I error. Given limited power, both significant (*p *< .05) and marginally significant (*p *< .10) results are discussed and effect sizes (Cohen's *d*) are presented throughout.

## 3 RESULTS

### 3.1 Descriptive statistics

Group‐specific means and standard deviations can be found in Tables 2 and 3. Correlations between study variables can be found in Table 4.

**Table 2:** Descriptive statistics and results of paired *t* tests at baseline

	High ERN *n* = 15	Low ERN *n* = 15	*t* (14)	*p*	*d*
	*M* (*SD*)	*M* (*SD*)			
Error‐Related Negativity	−8.81 (3.86)	1.05 (3.37)	−6.59***	<0.001	1.7
Cognitive Control					
Zoo task number correct go trials^a^	222.8 (34.5)	219.6 (52.0)	0.19	0.85	0.05
Zoo task number correct no‐go trials^a^	74.1 (12.4)	73.1 (17.4)	0.18	0.86	0.04
CBQ attention/inhibition composite^a^	10.4 (1.17)	9.62 (0.95)	1.7^†^	0.08	0.46
Emotion regulation/dysregulation					
CBQ negative affectivity^b^	9.88 (3.13)	12.3 (3.35)	−1.9^†^	0.05	0.50
Symptomotology					
Parent‐reported CBCL anxiety^b^	51.5 (3.08)	51.4 (3.52)	0.073	0.94	0.02
Parent‐reported CBCL internalizing^b^	43.2 (9.41)	45.7 (8.31)	−0.67	0.50	0.17
Parent‐reported CBCL externalizing^b^	43.5 (10.0)	48.0 (7.79)	−1.79^†^	0.07	0.45

^a^ Higher scores = better regulation.

^b^ Higher scores = more symptoms/problems.

^†^ *p *< .10.

* *p *< .05.

** *p *< .01.

*** *p *< .001.

**Table 3:** Descriptive statistics and results of paired *t* tests at follow‐up

	High ERN *n* = 15	Low ERN *n* = 15	*t* tests at follow‐up			*t* tests on residuals (follow‐up controlling for baseline)		
	*M* (*SD*)	*M* (*SD*)	*t* (14)	*p*	*d*	*t* (14)	*p*	*d*
Self‐regulation								
Dimensional Change card sort^a^	98.0 (11.2)	90.1 (9.51)	1.99*	0.047	0.50	1.55	0.12	0.40
Flanker task^a^	97.1 (16.7)	97.4 (10.5)	−0.05	0.96	0.01	−0.52	0.60	0.14
List sorting working memory task^a^	99.3 (9.79)	99.5 (11.6)	−0.09	0.93	0.02	−0.45	0.65	0.12
CBQ attention/inhibition composite^a^	11.0 (1.38)	9.27 (1.48)	3.43**	0.001	0.84	2.66**	0.008	0.59
BRIEF Behavior Regulation Index^b^	39.4 (9.72)	45.5 (8.20)	−1.81^†^	0.07	0.47	−0.98	0.33	0.25
BRIEF Metacognition Index^b^	67.4 (17.2)	75.3 (19.0)	−1.11	0.27	0.29	−0.15	0.88	0.01
BRIEF Global Executive Composite^b^	106.8 (25.9)	120.9 (24.7)	−1.38	0.17	0.36	−0.44	0.66	0.12
Emotion regulation/dysregulation								
Affect dysregulation^b^	3.07 (2.3)	6.07 (3.2)	−3.42**	0.001	0.88	−2.83**	0.005	0.66
ERC emotion regulation^a^	3.57 (0.34)	3.41 (0.49)	1.94^†^	0.05	0.61	1.08	0.28	0.22
ERC lability/negativity^b^	1.45 (0.31)	1.88 (0.36)	−3.22**	0.001	0.78	−2.50*	0.012	0.64
Symptomotology								
Child‐reported anxiety symptoms^b^	17.2 (9.9)	30.6 (11.5)	−3.89***	<0.001	1.01	−3.74***	<0.001	1.09
Child‐reported depression symptoms^b^	2.27 (2.2)	4.53 (2.2)	−4.02***	<0.001	1.04	−3.87***	<0.001	1.00
Parent‐reported CBCL anxiety^b^	52.3 (3.8)	53.6 (5.9)	−0.84	0.40	0.21	−1.01	0.31	0.26
Parent‐reported CBCL internalizing^b^	44.9 (8.8)	48.4 (8.2)	−1.32	0.19	0.34	−1.17	0.24	0.29
Parent‐reported CBCL externalizing^b^	44.1 (9.3)	55.7 (7.9)	−3.88***	<0.001	1.00	−3.94***	<0.001	1.10

^a^ Higher scores = better regulation.

^b^ Higher scores = more symptoms/problems.

^†^ *p *< .10.

* *p *< .05.

** *p *< .01.

*** *p *< .001.

**Table 4:** Correlations between study variables

	1	2	3	4	5	6	7	8	9	10	11	12	13	14	15	16	17	18	19	20	21	22	23
Baseline																							
1. ERN	1																						
2. Zoo go^a^	−0.20	1																					
3. Zoo no‐go^a^	−0.02	0.22	1																				
4. CC^a^	−0.28	−0.05	−0.09	1																			
5. NA^b^	**0.37**	0.03	−0.08	***−0.50***	1																		
6. ANX^b^	0.08	0.16	−0.05	0.05	***0.52***	1																	
7. INT^b^	**0.37**	0.01	0.10	−0.22	***0.56***	***0.72***	1																
8. EXT^b^	0.17	−0.06	−0.13	***−0.54***	**0.40**	0.22	**0.43**	1															
Follow‐up																							
9. DCCS^a^	**−0.36**	0.03	−0.06	0.22	−0.25	−0.17	−0.27	**−0.42**	1														
10. Flanker^a^	0.06	−0.03	−0.18	0.29	−0.04	−0.07	−0.20	−0.06	0.27	1													
11. WM^a^	−0.01	−0.08	−0.25	0.18	−0.19	0.02	−0.08	−0.11	0.35	−0.02	1												
12. CC^a^	***−0.50***	0.12	−0.06	***0.75***	−0.28	0.09	−0.11	**−0.39**	0.33	0.29	0.32	1											
13. BRI^b^	0.35	0.01	0.18	***−0.47***	0.36	0.21	**0.42**	***0.54***	−0.32	−0.02	−0.15	***−0.49***	1										
14. MI^b^	0.27	−0.01	0.22	***−0.54***	0.10	0.06	0.31	**0.41**	−0.28	−0.26	−0.26	***−0.72***	***0.72***	1									
15. GEC^b^	0.32	−0.01	0.22	***−0.55***	0.20	0.12	**0.37**	***0.49***	−0.32	−0.19	−0.24	***−0.69***	***0.87***	**0.97**	1								
16. ADS^b^	**0.44**	−0.05	0.20	−0.16	0.23	−0.17	−0.14	−0.13	−0.09	0.20	−0.10	−0.28	0.13	−0.01	0.04	1							
17. ER^a^	−0.10	0.15	−0.22	***0.51***	−0.25	0.14	−0.11	−0.25	0.17	0.05	0.15	**0.36**	***−0.52***	−0.35	**−0.44**	**−0.37**	1						
18. L/N^b^	***0.47***	−0.09	0.08	***−0.61***	***0.50***	0.14	**0.37**	0.**45**	−0.26	−0.04	0.03	***−0.57***	**0.79**	***0.51***	***0.65***	**0.43**	***−0.66***	1					
19. SCARED^b^	***0.50***	0.01	0.12	−0.35	0.35	−0.10	0.14	0.18	−0.19	−0.02	0.15	−0.21	0.22	0.03	0.10	***0.69***	***−0.47***	***0.52***	1				
20. CDI^b^	**0.43**	0.02	0.16	***−0.54***	0.25	−0.09	0.07	0.26	−0.01	0.09	−0.02	***−0.51***	**0.41**	**0.41**	**0.44**	***0.65***	***−0.55***	***0.60***	***0.72***	1			
21. ANX^b^	−0.01	0.11	−0.08	−0.14	0.18	−0.10	−0.14	0.22	−0.16	0.11	0.09	−0.04	**0.38**	0.17	0.26	0.34	−0.32	0.36	0.26	0.15	1		
22. INT^b^	0.22	−0.11	−0.10	−0.28	0.35	0.01	0.21	**0.39**	−0.21	−0.04	0.05	−0.21	**0.44**	0.34	**0.40**	0.31	**−0.44**	**0.42**	**0.43**	0.31	***0.78***	1	
23. EXT^b^	***0.54***	−0.05	0.02	***−0.75***	***0.55***	0.02	0.36	***0.71***	**−0.42**	−0.12	−0.25	***−0.68***	***0.69***	***0.54***	***0.63***	**0.38**	**−0.55**	***0.74***	***0.54***	***0.59***	0.35	***0.56***	1

Abbreviations: ADS, Affect Dysregulation Scale; ANX, Anxiety subscale from Child Behavior Checklist (CBCL); BRI , Behavior Regulation Index from Behavior Rating Inventory for Executive Functioning (BRIEF); CC,Cognitive Control composite from Child Behavior Questionnaire (CBQ); CDI, Child Depression Inventory; ER, Emotion Regulation subscale from the Emotion Regulation Checklist (ERC); ERN, Error‐related Negativity; EXT, Externalizing scale from CBCL; INT, Internalizing scale from CBCL; L/N, Lability/Negativity subscale from the ERC; MI, Metacognition Index from BRIEF GEC, Global Executive Composite from BRIEF; NA, Negative Affectivity; SCARED, Screen for Anxiety‐Related Disorders; WM, Working Memory.

**Bold** indicates *p *< .05, ***Bold and italics*** indicate *p *< .01, ***Bold, italic, and underlined*** indicate *p *< .001.

### 3.2 Between group differences at baseline

Results of paired *t* tests at baseline are found Table 2. Confirming our group selection, average ERN was significantly different between groups, *t*(14) = −6.59, *p *< .001, *d* = 1.7 (with the high‐amplitude group showing more negative mean level ERNs). Groups did not differ on behavioral performance on the EEG task (*p's *≥ .85), nor did they differ on anxiety (*p *= .94) or overall internalizing symptoms (*p *= .50) as rated by parents at baseline. The differences between groups on parent‐reported cognitive control (CBQ attention focus and inhibition; *t*(14) = 1.7, *p *= .08, *d *= 0.46), negative affectivity (*t*(14) = −1.9, *p *= .05, *d *= 0.50), and externalizing symptoms (*t*(14) = −1.79, *p *= .07, *d *= 0.45) were each marginally significant, with small to medium effect sizes. The high‐amplitude group tended to show better cognitive control, less negative affectivity, and fewer externalizing symptoms at baseline.

### 3.3 Between group differences at follow‐up

Results of paired *t* tests at follow‐up are found in Table 3. In terms of cognitive control, significant differences emerged between groups on the DCCS (*t*(14) = 1.99, *p *= .047, *d *= 0.50) and parent‐reported cognitive control (CBQ attention focus and inhibition; *t*(14) = 4.43, *p *= .001, *d *= 0.84) with the high‐amplitude group showing better executive functioning/cognitive control with medium to large effect sizes. There was an additional trend level difference on the behavior regulation index from the BRIEF (*t*(14) = −1.81, *p *= .07, *d *= 0.47), with the high‐amplitude group showing fewer behavior regulation difficulties. Score differences on the other BRIEF composites did not reach significance, but were in the same direction with small effect sizes (*d's* = 0.29–0.36). Performance on the working memory task and flanker task did not differ between groups at follow‐up (*p's *> .90).

In terms of emotion regulation/dysregulation, groups differed on child‐reported affect dysregulation (*t*(14) = −3.42, *p *= .001, *d *= 0.88) and parent‐reported lability/negativity (*t*(14) = −3.22, *p *= .001, *d *= 0.78) and emotion regulation (*t*(14) = 1.94, *p *= .05, *d *= 0.61). The high‐amplitude group reported less dysregulation (large effect size) and parents reported less lability/negativity and better emotion regulation (both medium effect sizes). For symptomatology, there were significant differences between groups for child‐reported anxiety (*t*(14) = −3.89, *p *< .001, *d* = 1.01) and depression (*t*(14 = −4.02, *p *< .001, *d* = 1.04), and for parent‐reported externalizing symptoms (*t*(14) = −3.88, *p *< .001, *d *= 0.1.0), with the high‐amplitude group showing fewer symptoms across measures (all large effect sizes). There were not significant differences between groups on parent‐reported anxiety (*p *= .40) or overall internalizing symptoms (*p *= .19); however, the direction of effect was consistent with other findings (small effect sizes).

### 3.4 Between group differences at follow‐up controlling for baseline

Using standardized residuals from the regression analyses described above in data analysis plan, differences in change from baseline to follow‐up were analyzed between groups. Significant differences at follow‐up, controlling for baseline, were found in parent‐reported cognitive control (*t*(14) = 2.66, *p *= .008, *d *= 0.59), child‐reported affect dysregulation (*t*(14) = −2.83, *p *= .005, *d *= 0.66), parent‐reported lability/negativity (*t*(14) = −2.50, *p *= .012, *d *= 0.64), child‐reported anxiety (*t*(14) = −3.74, *p *< .001, *d* = 1.09) and depression (*t*(14) = −3.87, *p *< .001, *d* = 1.0) symptoms, and parent‐reported externalizing symptoms (*t*(14) = −3.94, *p *< .001, *d* = 1.10). While the difference between groups on the DCCS controlling for baseline cognitive control did not achieve significance, it had a small to medium effect size (*d *= 0.40). Parent‐rated behavioral regulation (BRIEF), emotion regulation (ERC), anxiety (CBCL), and internalizing symptoms (CBCL) all showed non‐significant differences (*p's *~ 0.3), with small effect sizes (*d's *= 0.22‐0.29). Again, across measures, the high‐amplitude group displayed better regulation and fewer symptoms.

### 3.5 Mediation analysis

**Figure 1:**
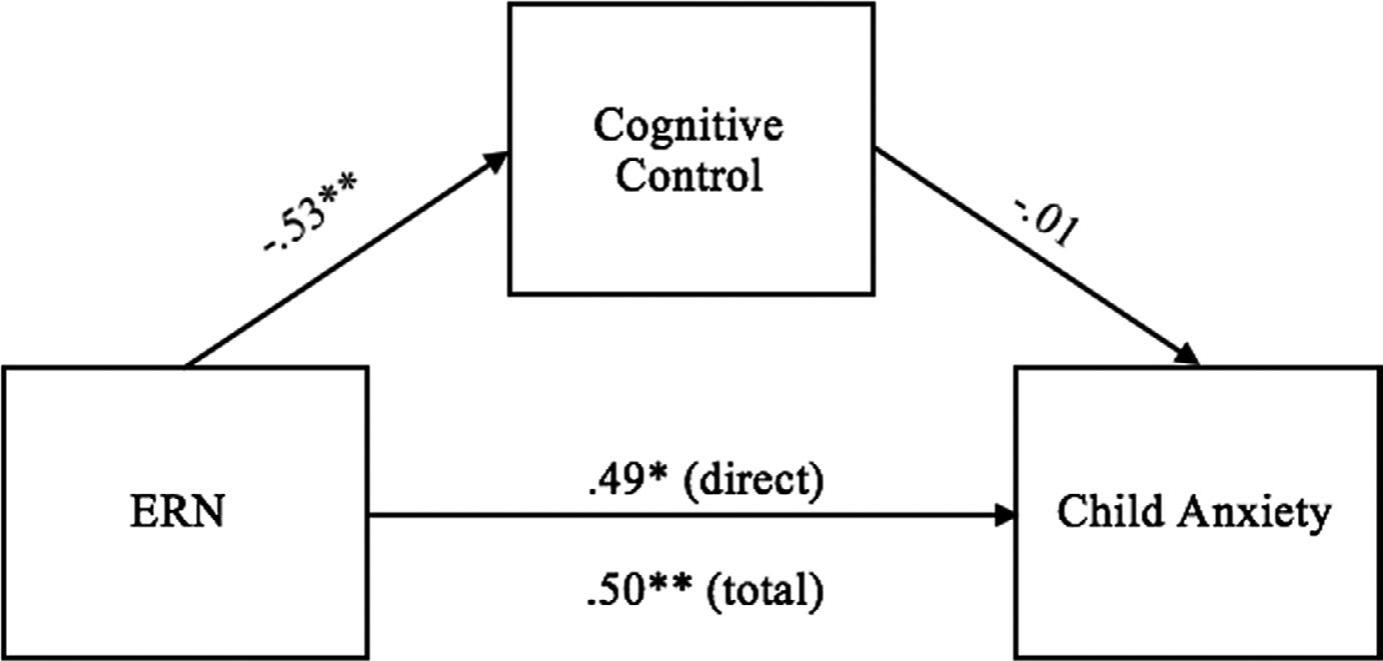
Model of the direct (significant) and indirect (non‐significant) effects of the ERN on child anxiety through self‐regulation. Values represent standardized beta coefficients. ^*^ *p* < .05. ^**^ *p* < .01

Two mediation models predicting between ERN and anxiety symptoms were run in PROCESS. The first used ERN at FCz as the predictor, follow‐up cognitive control composite (average of standardized DCCS, BRIEF BRI score [reversed], and CBQ attention focusing and inhibition) entered as the mediator, and follow‐up SCARED total score (child‐reported anxiety) as the outcome. The total effect of ERN on follow‐up anxiety symptoms was significant *b* = 1.02, *SE *= 0.33, *t* = 3.05, *p *= .005, such that a larger (i.e., more negative) ERN at baseline predicted fewer anxiety symptoms at follow‐up. The indirect effect through cognitive control was not significant, *b *= 0.01, *SE *= 0.10, Bootstrapped 95% CI = [−0.19–0.22]. The direct effect of ERN on anxiety also remained significant (see Figure 1).

**Figure 2:**
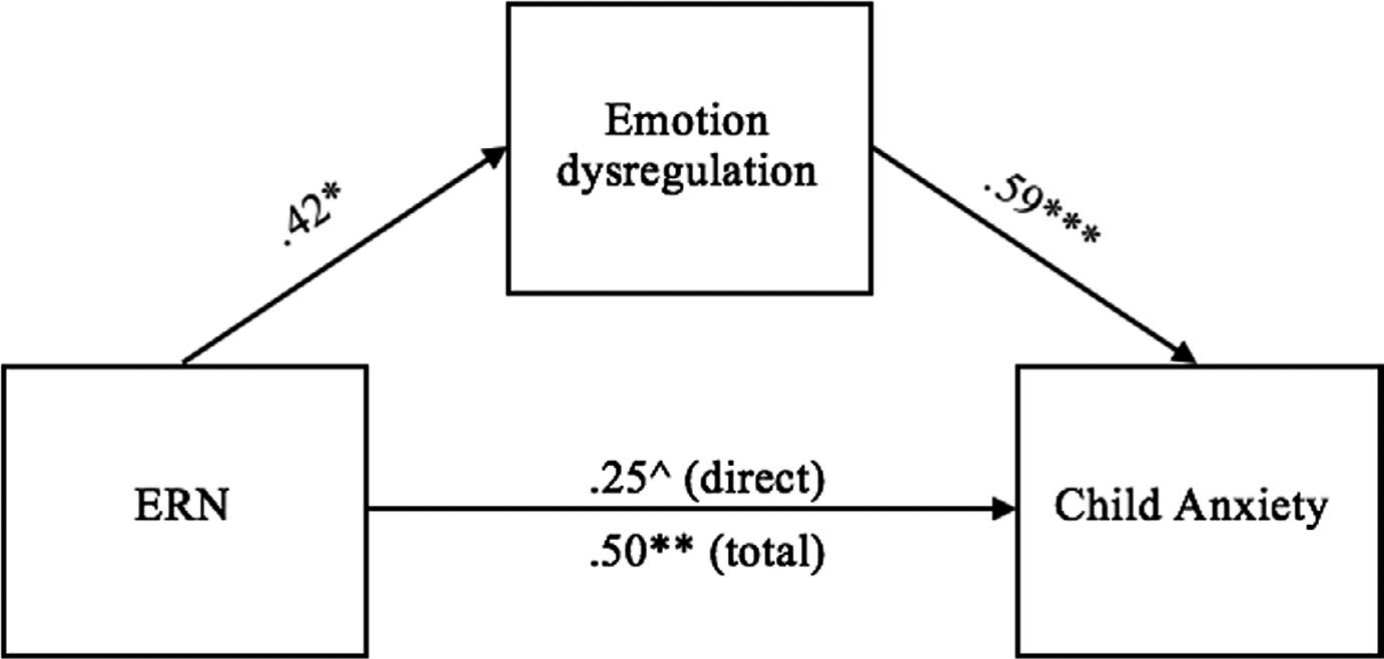
Model of the direct and indirect effects of the ERN on child anxiety through emotion dysregulation. Values represent standardized beta coefficients. ^^^ *p* < .10, ^*^ *p* < .05, ^**^ *p* < .01, ^***^ *p* < .001

The second model again used ERN at FCz as the predictor, but used an emotion dysregulation composite (average of child‐reported affect dysregulation, parent‐reported emotion regulation [reversed], and parent‐reported lability/negativity) as a mediator, and follow‐up SCARED total score (child‐reported anxiety) as the outcome. In this model, the indirect effect of ERN on anxiety symptoms through emotion dysregulation was significant, *b *= 0.24, *SE *= 0.09, Bootstrapped 95% CI = [.07–0.41], see Figure 2, such that the relationship between a larger baseline ERN and fewer follow‐up anxiety symptoms was mediated by greater capacity for emotion regulation at follow‐up. The direct effect became marginally significant with emotion dysregulation in the model.

**Figure 3:**
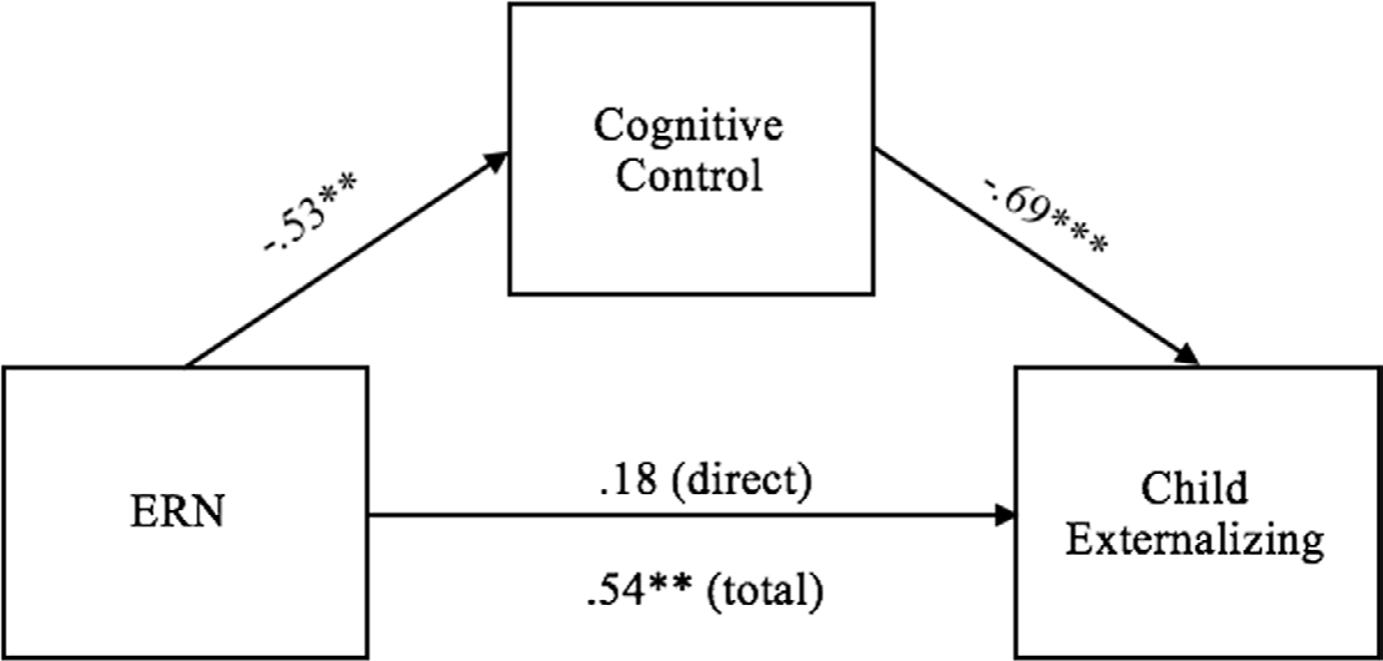
Model of the direct and indirect effects of the ERN on child externalizing problems through cognitive control. Values represent standardized beta coefficients. ^*^ *p* < .05. ^**^ *p* < .01, ^***^ *p* < .001

These two mediation models were repeated predicting to externalizing symptoms (parent report at follow‐up). In the first model (see Figure 3), the total effect of ERN on follow‐up externalizing symptoms was significant *b *= 0.54, *SE *= 0.16, *t* = 3.40, *p *= .002, such that a larger (i.e., more negative) ERN at baseline predicted fewer externalizing symptoms at follow‐up. The indirect effect through cognitive control was also significant, *b *= 0.36, *SE *= 0.11, Bootstrapped 95% CI = [.17–0.59]. The direct effect of ERN on externalizing problems was not significant.

**Figure 4:**
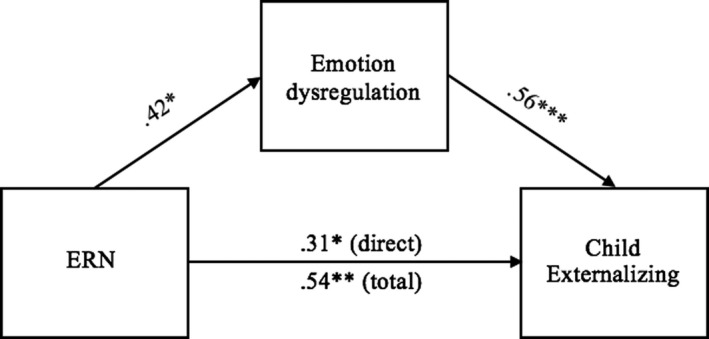
Model of the direct and indirect effects of the ERN on child externalizing problems through emotion dysregulation. Values represent standardized beta coefficients. ^*^ *p* < .05, ^**^ *p* < .01, ^***^ *p* < .001

In the final model (see Figure 4), mediation through emotion dysregulation was significant as well, *b *= 0.23, *SE *= 0.09, Bootstrapped 95% CI = [.06–0.42]. The direct effect of ERN on externalizing problems remained significant as well.

## 4 DISCUSSION

The current study examined the utility of the error‐related negativity (ERN) as a predictor of risk or resilience in young children. Our main aim was to elucidate the relationships between the ERN, anxiety symptoms, and cognitive control abilities over early to middle childhood using a longitudinal, extreme‐case design. In general, results demonstrate that the group of children with larger amplitude (more negative) ERNs measured in early childhood showed fewer anxiety symptoms, better cognitive control and better emotion regulation in middle childhood than the group of children with low‐amplitude ERNs. Specific results across different domains will be discussed below.

### 4.1 Differences at baseline

First, at baseline, when ERN and behavioral outcomes were measured concurrently, there was no difference between high and low ERN groups on anxiety, total internalizing symptoms, or on behavioral performance on the ERN‐eliciting inhibitory control task. There were marginally significant differences with small to medium effect sizes on parent‐reported cognitive control, negative affectivity, and externalizing symptoms. Children with low‐amplitude/blunted ERNs tended to show poorer cognitive control, more negative affectivity, and more externalizing symptoms. These results are relatively consistent with past literature. For example, Torpey et al., (2013) also found that young children with a smaller ERN displayed more negative affectivity measured by parent report on the CBQ. Meyer and Klein (2018) also found that blunted ERN was associated with externalizing symptomatology and reduced cognitive control (parent report CBQ). Past research has been inconsistent in regards to the relationship between ERN and behavioral performance on inhibitory control tasks, with some past studies (e.g., Santesso et al., 2006a; Torpey et al., 2012) showing such an association, but others (e.g., Santesso et al., 2006b; Wiersema et al., 2007) showing no association, which is in line with our results. This may be because the task is designed to elicit sufficient error trials to capture the ERN and thus may not be as sensitive to behavioral differences. Because parent report of cognitive control did show differences between groups, it may be that the ERN is more related to “hot” (emotion‐laden) cognitive control across day to day contexts, rather than the “cold” (strictly cognitive) nature of the computerized task. Further research should investigate this possibility.

The averages on parent‐reported anxiety between the two groups at baseline were nearly identical, which is in contrast to several recent studies showing a low‐amplitude ERN is concurrently associated with anxiety in young children (Lo et al., 2017; Meyer et al., 2012; Moser et al., 2015). These discrepant findings might be because our sample included a larger age range (5–7) than some prior studies and also demonstrated generally low levels of anxiety at baseline. However, our results at follow‐up are more in keeping with these past findings.

### 4.2 Differences at follow‐up

#### 4.2.1 Associations with cognitive control

At follow‐up, several additional differences appeared between groups. Executive function (inhibitory control/set shifting) measured on an objective task differed between groups, with the high‐amplitude group outperforming the low‐amplitude group. Interestingly, there were no differences on the other executive functioning tasks measuring working memory and attention. This may be because these tasks do not require the same level of conflict monitoring as the inhibitory control/set shifting task. Because the ERN has been most closely associated with conflict monitoring and control, it makes sense that tasks that rely heavily on this skill would show differences between groups. Interestingly, this is the age range when executive functions start to demonstrate a two‐factor rather than unitary factor structure (Brydges et al., 2014). Further research should explore the relations between ERN and different aspects of executive function over development.

Parents continued to rate children in the high‐amplitude group as higher on cognitive control, and additionally rated them as having better behavioral regulation on a parent report measure of executive functioning. The difference between groups on parent‐reported cognitive control (attention focus and inhibitory control) remained significant, even controlling for this measure at baseline. In other words, statistically controlling for baseline implies that the groups differed in their *change* in cognitive control as well, with that the high‐amplitude ERN group improving more than the low‐amplitude group over 1–2 years. This is consistent with and extends results from Grammer et al. (2018) who showed similar predictions over a 6‐month time period.

#### 4.2.2 Associations with emotion regulation

In terms of emotion regulation at follow‐up, parents rated children in the high‐amplitude group as having less lability/negativity and better emotion regulation than children in the low‐amplitude group. Further, children in the high‐amplitude group self‐reported significantly less affect dysregulation. These effects generally held even when accounting for negative affectivity reported by parents at baseline. Thus, larger ERN also could be considered a *predictor* of decreasing levels of negative affectivity over time. Given the associations between cognitive control (e.g. executive function and effortful control) and emotion regulation in past literature (e.g., Simonds et al., 2007) and in our study, it is not surprising that children in the high‐amplitude group were also better at regulating their emotions and experienced less lability/negativity and affect dysregulation over time.

#### 4.2.3 Associations with anxiety

Children with high‐amplitude ERN in early childhood also reported fewer anxiety symptoms at middle childhood, with a quite large effect size between groups, including when controlling for baseline parent‐reported anxiety severity. Parent report of this domain at follow‐up did not achieve statistical significance but was in the same direction as the self‐report results. The finding that larger ERN in early childhood predicts relatively fewer anxiety symptoms over time, whereas smaller ERN predicts worsening trajectories of anxiety compared to age‐mates is consistent with and extends the past research noted above. In particular, this result confirms the conclusion made by Meyer et al. (2018) that a blunted ERN in early childhood is consistent with a developmental trajectory biased toward risk for anxiety. These results suggest that in early childhood, a smaller ERN is a neural marker for risk for anxiety later in childhood.

#### 4.2.4 Associations with other psychopathology

We next tested how each group differed on depression and externalizing symptoms. Children in the high‐amplitude group also endorsed fewer depression symptoms than children in the low‐amplitude group. This finding held when controlling for parent‐reported internalizing symptoms at baseline. Previous studies have found a blunted ERN is related to depression symptoms in adolescence (Weinberg et al., 2016) and is associated with depressive disorder in children (Ladouceur et al., 2012). The current results extend this finding to younger children and also demonstrate the association across time. Parents also rated children in the high‐amplitude group as having fewer externalizing symptoms than children in the low‐amplitude group and this held when controlling for baseline level symptoms. This suggests that a blunted ERN is related not only to concurrent externalizing symptoms but also predicts change in externalizing symptoms over time.

#### 4.2.5 Mediational mechanisms

Finally, we explored whether cognitive control and emotion dysregulation mediated the association between larger baseline ERN and fewer symptoms at follow‐up. Our results show that emotion dysregulation acts as a mediator for the effect on anxiety while cognitive control does not. The only prior study to examine cognitive control, ERN, and anxiety in the same model, to our knowledge, is Meyer and Klein (2018). When predicting *to* ERN amplitude, they found that cognitive control mediated the relationship between fear and a decreased ERN, but not the relationship between shyness and an increased ERN. Further, they found that cognitive control did *not* account for the increased ERN in children with anxiety disorders, but shyness did mediate the association. We had expected that when predicting overtime cognitive control might account for the relationship between ERN and anxiety; however, our results were not consistent with this hypothesis. Rather, in our sample, emotion dysregulation accounted for the relationship between ERN and anxiety symptoms, indicating that it may be child ability to tolerate and manage distress that accounts for the relationship, rather than their cognitive control/executive function skills. Given the relationship between cognitive control and emotion regulation in young children found in past literature (e.g., Carlson & Wang, 2007) and in our study, it remains unclear how or if cognitive control abilities contribute to the relationship between the ERN, emotion regulation/distress tolerance, and anxiety. Given our small sample size and limited power, it is possible that a relationship between these variables exists but could not be detected. Further research with larger samples will be needed to elucidate these potential pathways.

Interestingly, both cognitive control and emotion dysregulation separately mediated the effect of the ERN on parent‐reported externalizing problems. This builds on Meyer and Klein’s (2018) finding that cognitive control mediated the association between externalizing symptoms and the ERN. Both cognitive control and affect regulation could be potential targets for intervention for children struggling with externalizing problems.

### 4.3 Limitations

This study has several limitations that should be noted. First and foremost, the modest sample size limits our statistical power. This is somewhat addressed by the extreme group design, which increases the chance of detecting an existing effect despite the small sample (Preacher et al., 2005). Our use of the extreme‐group approach was justified by circumstances such as the time‐consuming nature of several of our measures and the exploratory nature of some of our aims. However, it is important to note that extreme group designs tend to lead to inflated effect sizes, which should be interpreted with caution here.

Additionally, it is important to note that we examined symptomatology among a community sample, not in a group of children with clinically significant disorders. Past research has shown a differential association in early childhood between ERN and anxiety symptoms versus anxiety disorders. Further, the mean levels of anxiety and depression symptoms reported by the low‐amplitude group were still far under the clinical cut‐offs. Thus, future research should explore these relationships in a group of children with clinical disorders.

Also important to note is that findings were stronger for self‐reported symptomatology than for parent report. Differences between child and parent‐reported symptoms have been noted previously (e.g., Comer & Kendall, 2004) and others have pointed out the value of child self‐report especially when considering internalizing symptomatology that may not be obvious to an observer. Our results underscore the importance of self‐report for children, even as young as 7–9 years old. Finally, we did not have objective behavioral tests of emotion regulation. Future work should include these as well.

## 5 CONCLUSIONS

The current study extends our knowledge of the predictive utility of the ERN in early childhood. This time period may be particularly important for early detection of children at risk for trajectories of increasing symptomatology and could be used to identify children in need of preventative intervention.

## CONFLICT OF INTEREST

The authors have no conflicts of interest to disclose.

## AUTHOR CONTRIBUTIONS

Katherine L. Rosenblum, Maria Muzik, and Kate D. Fitzgerald contributed to initial study design, interpretation of data, and manuscript editing. Jamie M. Lawler contributed to study design, statistical analyses, and manuscript writing. Yanni Liu was responsible for EEG data acquisition and processing and manuscript writing. Jessica Hruschak contributed to study design, data acquisition, and data processing. Kristin Aho and Ka I Ip were responsible for literature searches and manuscript editing. Renee Lajiness‐O’Neill contributed to data interpretation and manuscript editing. All authors have contributed to and have approved the final manuscript.

### Peer Review

The peer review history for this article is available at https://publons.com/publon/10.1002/brb3.2008.

## Data Availability

The data that support the findings of this study are available from the corresponding author upon reasonable request (Lawler et al., 2020).
